# Progress and challenges in directing the differentiation of human iPSCs into spinal motor neurons

**DOI:** 10.3389/fcell.2022.1089970

**Published:** 2023-01-05

**Authors:** Cristina Marisol Castillo Bautista, Jared Sterneckert

**Affiliations:** ^1^ Center for Regenerative Therapies TU Dresden (CRTD), Technische Universität (TU) Dresden, Dresden, Germany; ^2^ Medical Faculty Carl Gustav Carus of TU Dresden, Dresden, Germany

**Keywords:** assembloids, neurodegenerative diseasaes, organoids, motor neurons, iPS cells, induced pluripotent stem cells, directed differentiation of pluripotent stem cells

## Abstract

Motor neuron (MN) diseases, including amyotrophic lateral sclerosis, progressive bulbar palsy, primary lateral sclerosis and spinal muscular atrophy, cause progressive paralysis and, in many cases, death. A better understanding of the molecular mechanisms of pathogenesis is urgently needed to identify more effective therapies. However, studying MNs has been extremely difficult because they are inaccessible in the spinal cord. Induced pluripotent stem cells (iPSCs) can generate a theoretically limitless number of MNs from a specific patient, making them powerful tools for studying MN diseases. However, to reach their potential, iPSCs need to be directed to efficiently differentiate into functional MNs. Here, we review the reported differentiation protocols for spinal MNs, including induction with small molecules, expression of lineage-specific transcription factors, 2-dimensional and 3-dimensional cultures, as well as the implementation of microfluidics devices and co-cultures with other cell types, including skeletal muscle. We will summarize the advantages and disadvantages of each strategy. In addition, we will provide insights into how to address some of the remaining challenges, including reproducibly obtaining mature and aged MNs.

## Introduction

Amyotrophic lateral sclerosis, progressive bulbar palsy, primary lateral sclerosis, and spinal muscular atrophy are motor neuron (MN) diseases characterized by progressive degeneration of MNs, resulting in the loss of voluntary and involuntary movement (Vianello et al., 2022). Since the molecular mechanisms of pathogenesis of MN diseases are not well understood, it has been extremely difficult to identify effective treatments.

MNs control movement by integrating signals from the brain and sensory systems (Swetenburg et al., 2017). Upper MNs are located within the motor cortex of the brain and lower MNs are located within the brainstem as well as spinal cord. Lower MNs include branchial MNs, which are located in the brainstem and innervate the face and neck, visceral MNs, which are part of the autonomic nervous system and innervate smooth muscle, as well as somatic MNs, which are mostly located within the spinal cord and innervate skeletal muscle in the periphery. Our review focuses on spinal MNs innervating skeletal muscle (e.g., somatic MNs). Spinal MNs are organized along the rostrocaudal axis into motor columns, including the median motor column, the lateral motor column, the hypaxial motor column, the preganglionic column, the spinal accessory column, and the phrenic motor column, each of which innervate distinct peripheral regions (Stifani, 2014). For additional information about each motor column and other MN subtypes, please see Stifani. (2014).

One of the most significant challenges in studying these diseases is that patient MNs are located within the brain (upper MNs) and spinal cord (lower MNs), making them only accessible in post-mortem tissue. However, the MNs of most interest for protecting are not available for analysis in post-mortem tissue because they degenerate during the disease course. Thus, the earliest stages of pathogenesis, which might be amenable to a therapeutic intervention, occur while these MNs are hidden from view. Therefore, another model system is needed to recapitulate early MN pathogenesis using living human MNs.

Induced pluripotent stem cells (iPSCs) are a powerful tool for understanding human MN biology and disease pathogenesis, particularly in their earliest stages. Takahashi and Yamanaka demonstrated that the expression of four transcription factors, OCT4, SOX2, KLF4, and C-MYC, reprograms somatic cells into iPSCs (Takahashi & Yamanaka, 2006) that have the ability to undergo theoretically limitless self-renewal and to differentiate into all somatic cell types as well as germ cells (Takahashi et al., 2007). Combining these two properties, researchers use iPSCs to generate vast quantities of human MNs containing the same genetic polymorphisms as the donor patient. By comparing iPSC-derived MNs from patients against those from unaffected individuals or gene-corrected controls, it is possible to identify molecular mechanisms of pathogenesis (Bhinge et al., 2017; Ho et al., 2021). Of interest, the scalable production of iPSC-derived MNs is compatible with high-throughput screening to identify potential therapeutics to protect MNs against disease pathogenesis (Fujimori et al., 2018; Marrone et al., 2018; Fang et al., 2019). Thus, iPSCs are poised to have an enormous impact on our understanding and treatment of MN diseases.

Reaching their transformative potential requires efficient and consistent differentiation of iPSCs into functional MNs. Many labs have contributed to the development of increasingly sophisticated differentiation protocols to obtain iPSC-derived MNs, including the use of specific small molecules to recapitulate developmental morphogen signals (Dimos et al., 2008; Amoroso et al., 2013; Du et al., 2015), expression of lineage-specific transcription factors (Hester et al., 2011; Goparaju et al., 2017; De Santis et al., 2018), as well as the use of 3-dimensional (3-D) cultures, including organoids (Chandrasekaran et al., 2017; Ogura et al., 2018; Besser et al., 2020). Several differentiation protocols focus specifically on MN axons by implementing microfluidic devices within their differentiation protocol (Osaki et al., 2018b; Mo et al., 2020; Spijkers et al., 2021; Stoklund Dittlau et al., 2021; Hagemann et al., 2022). In addition, multiple investigators utilize co-cultures with other cell types, including skeletal muscle, to reconstitute a functional MN circuit (Osaki et al., 2018c; Bellmann et al., 2019; Lin et al., 2020). Despite these significant advances, several critical points still require improvement, including, reducing variability in the yield, and improving maturation in order to more accurately recapitulate MN function.

It is important to match the research question with the appropriate model. Many protocols, particularly in 2-dimensional cultures, focus on obtaining homogeneous populations of MNs, and these cultures are particularly useful for studying the development of MNs as well as cell-autonomous disease pathology. However, a different strategy would be needed to understand more complex systems such as motor circuits and non-cell autonomous disease pathogenesis, including prion-like spreading, which would require reproducible heterogeneous cultures. Here, we review reported differentiation protocols for obtaining spinal MNs, summarize the advantages and disadvantages of each strategy, and discuss possible solutions for the remaining challenges.

## Using small molecules to direct the differentiation of iPSCs into MNs

**FIGURE 1:**
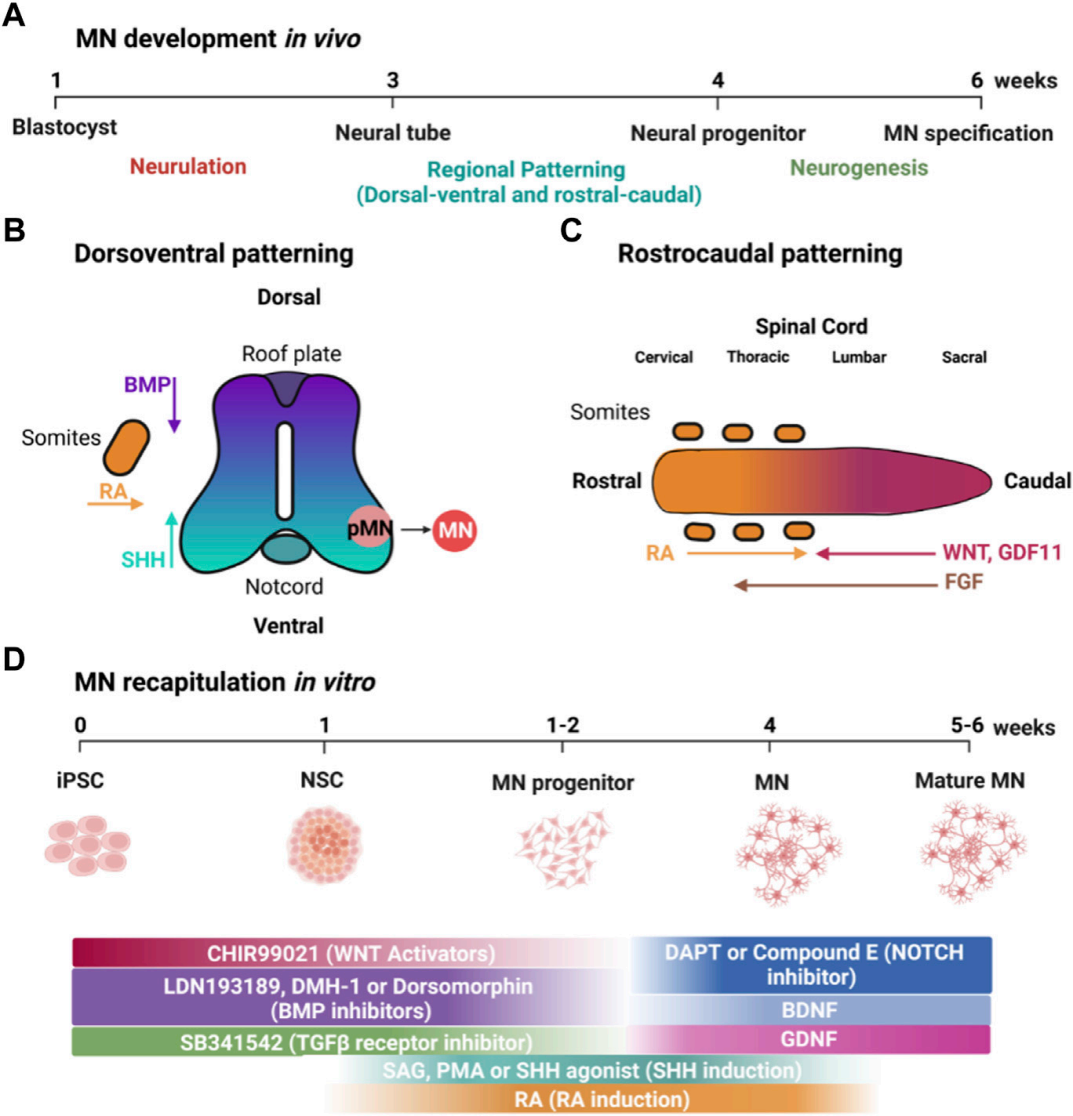
MN development *in vivo* and its recapitulation *in vitro* using small molecules. **(A)** Timeline of MN development *in vivo*. **(B)** Patterning along the dorsoventral axis is regulated by BMP and SHH produced by the roof and floor plates, respectively, as well as RA from adjacent somites. **(C)** Patterning of the rostrocaudal axis is regulated by WNT, FGF, GDF11 and RA. **(D)** Timeline showing how small molecules can be used to recapitulate developmental factors that specify MN development. Created with BioRender.com.

During development, multiple carefully coordinated signaling factors specify MN fate. Many protocols (summarized in Table 1) direct the differentiation of iPSCs into MNs using specific small molecules to recapitulate these signaling factors *in vitro* (Figure 1). This approach was first pioneered using mouse embryonic stem cells (mESCs) (Wichterle & Peljto, 2008), which provided invaluable insights for the subsequent differentiation of human pluripotent stem cells, including human embryonic stem cells (hESCs) and iPSCs to MNs (Wichterle et al., 2002; Li et al., 2005; Lee et al., 2007).

**TABLE 1:** Protocols using small molecules to direct the differentiation of human iPSCs into MNs.

Year	Small molecules	Duration (days)	MN efficiency (%)	References
2008	RA, SHH, BDNF, GDNF, CTNF	30	20	Dimos et al. (2008)
2009	RA, SHH, BDNF, GDNF, IGF-1	35	>40	Hu & Zhang, (2009)
2009	RA, PMA, GDNF, BDNF, CTNF	48–62	>35	Karumbayaram et al. (2009)
2012	SB, Dorsomorphin, RA, PMA, BDNF, GDNF, forskolin	71	>10	Bilican et al. (2012)
2013	LDN, SB, RA, SHH, BDNF, GDNF, CNTF	31	30	Amoroso et al. (2013)
2013	RA, PMA, GDNF, BDNF	31	40	Sareen et al. (2013)
2014	LDN, SB, RA, SAG, PMA, BDNF, GDNF, CNTF	31	>30	Grunseich et al. (2014)
2013	Dorsomorphin, RA, SHH, BDNF, GDNF, IGF-1	20	70	Qu et al. (2014)
2015	Dorsomorphin, SB, BIO (GSK inhibitor), RA, PMA, c-AMP, BDNF, GDNF, IGF-1	28–42	>60	Shimojo et al. (2015)
2015	SB, LDN, RA, SAG, CHIR, DAPT	25	70	Maury et al. (2015)
2015	CHIR, DMH-1, SB, RA, PMA, BDNF, GDNF, CNTF, IGF-1, Compound E	30	95	Du et al. (2015)
2016	RA, SHH, c-AMP, BDNF, GDNF, IGF-1	21–30	40	Dafinca et al. (2016)
2016	LDN, SB, RA, PMA, CHIR, c-AMP, BDNF, GDNF, IGF-1	28	80	Hu et al. (2016)
2018	CHIR, DMH-1, SB, RA, PMA, BDNF, GDNF, CNTF, IGF-1, Compound E	18–53	60	Sances et al. (2018)
2018	CHIR, Dorsomorphin, Compound E, SHH, SAG, PMA, RA, CTNF, BDNF, GNDF, NT-3	21	73	Bianchi et al. (2018)
2018	SB, CHIR, Dosormorphin, RA, PMA, DAPT, db-cAMP, BDNF, GDNF, IGF-1	40	70	Fujimori et al. (2018)
2018	LDN, SB, RA, SHH, BDNF, GDNF, CNTF	27	20	Bossolasco et al. (2018)
2019	SB, Dorsomorphin, RA, PMA, BDNF, GDNF, forskolin	28–42	>90	Bax et al. (2019)
2020	CHIR, DMH-1, SB, RA, PMA, BDNF, GDNF, CNTF, IGF-1, Compound E	28	67	Thiry et al. (2020)
2020	Dorsomorphin, FGF2, Noggin, SB, RA, SHH, BDNF, GDNF, IGF-1	20	80	Faye et al. (2020)
2020	SB, LDN, RA, SAG, CHIR, DAPT	29	>90	Garcia-Diaz et al. (2020)
2021	LDN, SB, IWR1e, CHIR, RA, PMA, DAPT	28–42	16	Sato et al. (2021)
2021	RA, PMA, BDNF, GDNF, IGF-1, c-AMP	28–52	>90	Cutarelli et al. (2021)

iPSCs closely resemble hESCs, which are derived from the inner cell mass of blastocysts, which form the epiblast that will undergo gastrulation to form three germ layers: ectoderm, mesoderm, and endoderm (Swetenburg et al., 2017). The neural plate (Figure 1A) is formed from the dorsal ectoderm through the inhibition of transforming growth factor β (TGF-β) and bone morphogenetic proteins (BMP) signaling together, termed dual-SMAD inhibition (Chambers et al., 2009). Dual-SMAD blocks the endodermal and mesodermal cell fates and directs instead from ectoderm toward differentiation into neural stem cells (Jessell & Sanes, 2000). In a similar fashion, iPSCs form neuroepithelial cells using small molecules such as SB431542 (SB) and LDN193189 (LDN), which inhibit TGF-β and BMP signaling, respectively, and is sometimes referred to as dual-SMAD inhibition (Chambers et al., 2009) (Figure 1D).

During neurulation, the neural plate folds to form the neural tube. MNs form in the ventral neural tube, and, thus, dorsoventral patterning plays an essential role in inducing MN formation (Figure 1B). Retinoic acid (RA), secreted by adjacent somites, is essential in developmental dorsoventral patterning and specification of spinal MNs (Puttagunta & Di Giovanni, 2011). Ventral fate is specified by a gradient of sonic hedgehog (SHH) emanating from the notochord and floor plate (Liem et al., 2000). In contrast, dorsal fate is patterned by an opposing gradient of BMP signaling from the roof plate, forming the dorsoventral axis along the spinal cord (Hor & Ng, 2020). *In vitro*, iPSCs are similarly ventralized using SHH signaling (Figure 1D). Although some protocols utilize recombinant SHH protein, it is more frequent to use small molecules such as RA in combination with purmorphamine (PMA) or smoothened agonist (SAG), which activate the SHH receptor (Wichterle et al., 2002; Amoroso et al., 2013) and are more affordable and compatible with large-scale experiments.

Rostrocaudal patterning also plays an important role in the specification of MNs in the developing neural tube (Figure 1C) (Stifani, 2014). WNT signaling is essential for the specification of caudal identity (Rosso & Inestrosa, 2013). WNT ligands are expressed in the primitive streak and roof plate, but, at later stages, are also expressed in multiple dorsal neural progenitors (Megason & McMahon, 2002). FGFs and GDF11 are also important inducers of caudal identity (Liu et al., 2001). GDF11 enhances the ability of FGFs to induce a caudal fate as marked by the expression of HOX transcription factors (e.g., the “HOX code”). In addition, RA, secreted by adjacent somites, is important for inducing spinal cord identity in the developing neural tube (Davis-Dusenbery et al., 2014). In a similar fashion, caudalization of iPSC-derived neuroepithelial cells is induced by small molecules such as CHIR-99021 (CHIR), which activates canonical WNT signaling *via* inhibition of glycogen synthase kinase 3 (GSK3), in combination with RA (Maury et al., 2015) (Figure 1D). Lippmann and colleagues were able to direct the differentiation of human PSCs into different regions of the hindbrain and spinal cord using a combination of WNT, FGF, RA, and GDF11 (Lippmann et al., 2015). They found that GDF11 facilitates lumbosacral patterning. Lim and colleagues directed iPSC differentiation using different rostrocaudal MN subtypes in a spatially resolved manner with controlled gradients of RA and GDF11 in the presence of a fixed concentration of PMA (1 μM) (Lim et al., 2019). RA and GDF11 guide spatial differentiation by promoting the rostralization of MNs in the brachial region and caudalization in the thoracic and lumbar regions.

NOTCH signaling promotes proliferation of neural progenitors. Correspondingly, small molecules such as DAPT and compound E, which inhibit the NOTCH signaling pathway promote maturation of MNs *in vitro* (Du et al., 2015; Venkatesh et al., 2017). Moreover, neurotrophic factors, including insulin-like growth factor-1 (IGF-1), glial-derived neurotrophic factor (GDNF), and brain-derived neurotrophic factor (BDNF) promote MN survival for cultivation *in vitro* (Li et al., 2005; Hu & Zhang, 2009; Burkhardt et al., 2013).

## Small changes in small molecule usage have large effects on differentiation efficiency

Correct specification of MNs during development requires application of each of these signaling factors at a precise time and concentration. Thus, each small molecule used to differentiate iPSCs must be optimized to obtain the most efficient yield of MNs. The first report on iPSCs to produce patient-specific MNs was in 2008 and used a combination of RA and SHH protein for 45 days, resulting in a yield of 20% MNs (Dimos et al., 2008). Initial efforts to increase the yield of MNs focused on the signaling factors used for caudalization and ventralization. Introducing RA and SHH earlier and over a more extended period increased the differentiation efficiency to 50% (Hu & Zhang, 2009). Amoroso and colleagues used a higher concentration of RA in combination with SAG and PMA, resulting in an accelerated MN differentiation within 30 days, but with only 30% MNs (Amoroso et al., 2013). Many additional studies report efforts to further improve MN yield by modifying the timing and concentration of small molecules to induce caudalization and ventralization. Unfortunately, the maximal yield using this approach was about 50% (Patani et al., 2011; Bilican et al., 2012; Sareen et al., 2013; Grunseich et al., 2014). This suggests that optimization of other factors is required for increased differentiation efficacy.

Indeed, focusing on the small molecules used for neural induction significantly improved MN differentiation efficiency. In 2014, Qu et al. (2014) and colleagues used Dorsomorphin (also known as Compound C), which blocks both Activin and BMP pathways, during the first 6 days of neural induction in iPSCs, resulting in 70% MNs after 20 days. Moreover, they found that the earlier addition of RA (day 2) or later (day 6) during neural induction results in a weak MN fate specification. In contrast, RA addition at day 3 induces MN differentiation with very high efficacy.

Modifying the small molecules used for dual-SMAD inhibition achieved the highest reported MN yield (Du et al., 2015). The BMP inhibitor DMH1, in combination with the TGF-β inhibitor SB as well as WNT agonist CHIR achieved 95% MNs in 28 days, one of the highest yields of MNs reported. In addition, the intermediate MN progenitors could be expanded as well as cryopreserved. One team attempted to improve MN differentiation efficiency by combining dual-SMAD inhibition with γ-secretase inhibitors and WNT activators (Bianchi et al., 2018). After 21 days, about 73% of cells expressed MN markers and, after 5 weeks, were electrophysiologically functional.

## Challenges with using small molecules that require further optimization

Despite considerable success with using small molecules to recapitulate developmental cues and direct MN differentiation, significant challenges remain, including achieving reproducible yields and homogeneity in the resulting cultures. Applying the same differentiation protocol to different iPSC lines frequently results in varying differentiation efficiencies (Hu et al., 2010; Volpato et al., 2018). Even more frustratingly, applying a protocol to different passages or subclones of the same iPSC line can also result in different percentages of MNs obtained (Koehler et al., 2011; Cantor et al., 2022). We find that following reported protocols using our own iPSCs results in a significantly lower yield, suggesting a need to fine-tune differentiation protocols for individual cell lines. The presence of non-MNs can significantly complicate downstream experiments, including high-throughput microscopy screens or “-omics” applications (Fernandopulle et al., 2018). The molecular mechanisms driving these differences in the differentiation between individual iPSC lines are not clear. One possibility is that iPSC lines secrete their own unique signature of autocrine factors and those changes in autocrine signaling factors could affect MN differentiation efficiency. Interestingly, male and female lines show significant differences in differentiation efficacy (Boulting et al., 2011).

Another factor that has to be considered is that the variability of undifferentiated iPSCs could be linked to differences in genetic polymorphisms (Burrows et al., 2016), The Human Induced Pluripotent Stem Cells Initiative reported that 5%–46% of the variation in undifferentiated iPSC cell phenotypes is due to inter-individual differences (Kilpinen et al., 2017). In another study, Carcamo-Orive et al. (2017) and colleagues observed genetic and non-genetic determinants of the heterogeneity of iPSC lines through analysis of transcriptional variability. They analyzed the gene expression variability in 317 iPSC lines from 101 individuals. Around 50% of genome-wide expression variability could be explained by variation across individuals, and they identified a set of expression quantitative trait loci that contribute to this variation. To reduce variation, rigorous standardization of iPSC reprogramming and differentiation must occur (Volpato & Webber, 2020).

It is theoretically possible to continually optimize the small molecules used for each iPSC line and passage. For example, Maury et al. (2015) and colleagues demonstrate one approach for optimizing each factor to obtain different types of MNs. Nevertheless, this is clearly cumbersome. Thus, purification steps, such as magnetic-activated cell sorting (Garcia-Diaz et al., 2020) have arisen to isolate differentiated MNs from heterogeneous cultures, reducing the dependence on ongoing optimization to obtain pure cultures of MNs.

One important disadvantage of using small molecules to recapitulate MN specification in a dish is the amount of time required. Most small molecule-based differentiation protocols require weeks or even months (see Table 1), which clearly limits the number as well as the type of experiments that can be performed. In addition, there are data suggesting that MN cultures most closely resemble fetal MNs (Ho et al., 2016), which is clearly a limiting factor when using MNs to model age-associated MN diseases, such as amyotrophic lateral sclerosis. Long-term cultivation of heterogeneous cultures can also pose technical challenges. For example, if even a few proliferative cells contaminate the initial culture of MNs, then these proliferative cells can quickly outcompete post-mitotic MNs during long-term cultivation.

Another challenge is the detachment of the MNs in long-term culture. In our experience, MNs in culture plated in Matrigel or poly-L-ornithine and laminin for up to 2 months start detaching. For this reason, the use of other coating substrates, such as dendritic polyglycerol amine (Thiry et al., 2022), has been suggested to improve conditions for long-term cultures of iPSC-derived MNs.

Thus, it has been difficult to achieve the goal of a rapid differentiation protocol with a very high yield of MNs from different iPSCs over many passages using small molecules. The long culture times and heterogeneity that has resulted has led investigators to explore other approaches, including the use of expression of transcription factors found in developing MNs*.*

## Using transcription factors to direct the differentiation of iPSCs into MNs

**FIGURE 2:**
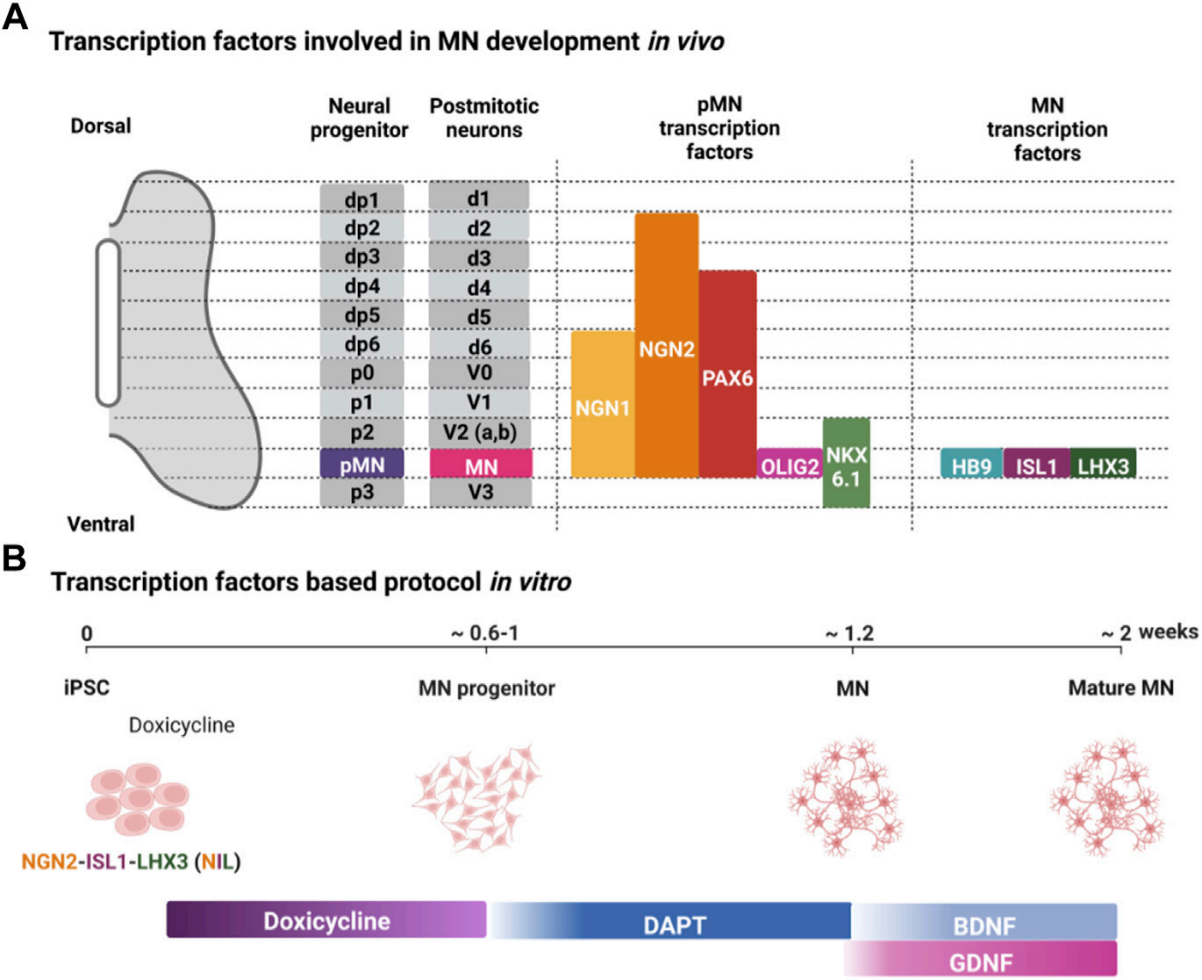
Transcription factors regulating MN development *in vivo* and their use in differentiating iPSCs into MNs *in vitro*. **(A)** Transcription factors expressed in the MN progenitors (pMN) and post-mitotic MNs (MN) in the developing neural tube. **(B)** Representative protocol for using expression of specific transcription factors to direct the differentiation of iPSCs into MNs. Created with BioRender.com.

Signaling factors in the ventral neural tube specify developing MN progenitors by inducing the expression of specific transcription factors (Figure 2A). SHH specifies MN fate *via* the expression of the class II transcription factors OLIG2 and NKX6.1, and RA promotes the expression of the class I transcription factor PAX6 (Tanabe et al., 1998; Briscoe et al., 2000; Novitch et al., 2001). OLIG2 regulates expression of NGN2 in MN progenitors, and NGN2 expression promotes cell cycle exit of post-mitotic MNs that express the transcription factors HB9 (also known as MNX1), ISL1, and LHX3 (Novitch et al., 2001). These transcription factors are determinants of MN identity and are initially expressed in all post-mitotic MNs whose axons exit the spinal cord *via* the ventral root (Santiago & Bashaw, 2014).

Since specific transcription factors are expressed downstream of developmental signaling factors, ectopic expression of these transcription factors should direct differentiation of MNs and reduce heterogeneity arising from differences in autocrine signaling factors. Mouse ESCs provide some of the first proof-of-principle for using transcription factors to direct MN differentiation (Mazzoni et al., 2013). Two doxycycline-inducible mESC lines were generated: one harboring a polycistronic expression construct NGN2 + ISL1 + LHX3 (NIL) for spinal MNs, and NGN2 + ISL1 + PHOX2A (NIP) for cranial MNs. In 6 days, using NIL, mESCs formed spinal MNs with almost 80% efficiency. This efficiency is strikingly higher than what has been achieved with many small molecule-based approaches.

Inspired by the striking results using mESCs, transcription factors have been extensively used to direct hESCs and iPSCs into MNs (Summarized in Table 2). Hester and colleagues used adenovirus to deliver and express NIL in hESC- and iPSC-derived neural progenitor cells, resulting in more than 70% MNs after 11–13 days (Hester et al., 2011). Another study used Sendai virus to express NIL in undifferentiated iPSCs rather than in neural progenitors, making it a more direct approach, and yielded 30% MNs after 14 days (Goto et al., 2017). Interestingly, expression of all three NIL transcription factors was required for MN differentiation. No MNs were obtained after ectopic expression of any one transcription factor, nor when NGN2 was expressed in combination with ISL1.

**TABLE 2:** Protocols using transcription factor expression to direct the differentiation of human iPSCs into MNs.

Year	Delivery system	Transcription factors	Duration (days)	MN efficiency (%)	References
2011	Adenovirus	NGN2, ISL-1, LHX3	11	72	Hester et al. (2011)
2017	Sendai virus	NGN2, ISL-1, LHX3	14	30	Goto et al. (2017)
2017	Synthetic mRNA	NGN1, NGN2, NGN3, ND1, ND2, ND4	14	94	Goparaju et al. (2017)
		ND6			
2018	pMx retroviral vector	NGN2, ISL1, LHC3, ND1, BRN2, ASCL1, MYT1L	15	70–80	Shi et al., 2018, Shi et al. (2018)
2018	AAVS1 locus	NGN2, ISL-1, LHX3	14	90–100	Fernandopulle et al. (2018)
2018	PiggyBac	NGN2, ISL-1, LHX3 and NGN2 + ISL1 + PHOX2A	13	90	De Santis et al. (2018)

## Efforts to further optimize the use of MN transcription factors

Although these reports using transcription factors to direct MN differentiation are very impressive, there is still a need for further optimization. For example, viruses can be cumbersome to use because of the need for packaging and cultures must be infected before each experiment. In addition, the infection conditions need optimization for each individual cell line, and cells within one culture can be stochastically infected with a different number of viruses, potentially resulting in heterogeneous transgene expression. Also, viral infection is potentially toxic and, depending on the virus used, there could be a risk of insertional mutagenesis.

One possible approach to avoid the use of viruses is synthetic mRNAs. Synthetic mRNAs encoding specific transcription factors can be transfected into iPSCs, potentially allowing for robust, footprint-free dose-dependent expression. Goparaju and colleagues found syn-mRNAs encoding NGN1, NGN2, NGN3, NEUROD1, and NEUROD2 showed a strong neural induction by Day 5 (Goparaju et al., 2017). Moreover, by day 14, about 95% of TUBB3-positive neurons were MNs. However, similar to viruses, mRNAs are cumbersome to use because they require synthesis. Also, transfection can be toxic and result in heterogeneous expression of the transgenic protein.

De Santis et al. (2018) and colleagues used a PiggyBac transposable vector derived from epB-Bsd-TT, which was integrated into iPSCs. Spinal and cranial MNs were obtained using integrated PiggyBac vectors to express NIL and NIP, respectively. This approach resulted in MNs considerably quicker than any report using small molecules. The post-mitotic MN markers TUBB3, ISL1, and ChAT were detected within 3 days of differentiation. By day 5, Hb9 expression was increased together with LHX3 or PHOX2A for spinal and cranial MNs, respectively. Efficacy for both types of MNs was more than 90%. Moreover, about 50% displayed spontaneous glutamatergic post-synaptic currents after 13 days of differentiation (Figure 2B) (De Santis et al., 2018). A similar approach is to use inducible NIL vectors stably integrated into iPSCs at a safe harbor locus such as AAVS1 (Fernandopulle et al., 2018). Fernandopulle and colleagues showed expression of MN markers at 14 days. It is important to note that this transcription factor approach seems to be strikingly more efficient than small molecules. At its most efficient, small molecules direct the differentiation of iPSCs into MNs with up to 95% efficiency after 28 days. Thus, using transcription factors seems to reduce the time required by about half.

Unfortunately, similar to the small molecule-based approach, there is still an issue with immaturity of the resulting MNs. Although expression of transcription factors such as NIL efficiently direct differentiation into MNs, these transcription factors are expressed in MNs during development. It is likely that other transcriptional programs are needed for MN maturation and function. Thus, continued expression of early embryonic MN transcription factors might reinforce an immature MN fate and inhibit further maturation. One way to overcome this issue is to avoid permanently integrating a construct into the genomic DNA, and, instead, transiently transfecting synthetic mRNAs. Goparaju et al. (2017) and colleagues provided proof of principle for this approach by demonstrating that a cocktail of five synthetic transcription factor mRNAs directed the differentiation of human PSCs into neurons with over 80% efficiency, of which over 90% were MNs. Additional work is needed to assess if this approach is better suited to generating mature MNs compared to the use of vectors integrated into the genome.

In the future, it might be possible to modify the combination of TFs to more directly program the differentiation of mature MNs. Patel et al. (2022) and colleagues evaluated the transcriptional dynamics of mouse spinal MNs and identified four candidates as regulators of MN maturation: NFI transcription factors, steroid hormone receptors, AP-1, and Mef2. In addition, they found that, after specification, the selector transcription factor LHX3 is downregulated in most MNs and the regulatory elements controlled jointly by ISL1 and LHX3 become decommissioned. It might be possible, in the future, to use information from this and similar studies to efficiently differentiate iPSCs into mature MNs.

Meanwhile, it is known that during development, MNs interact with many other cell types. Thus, MN maturation might require the presence of other cells such as glial cells, including oligodendrocytes, astrocytes, and microglia, as well as skeletal muscle cells. Moreover, these interactions often take place in a spatially separated manner–e.g., Soma and dendrites with astroglia and axons with Schwann cells and skeletal muscle in the periphery. In the following section, we describe the techniques and devices used to develop co-cultures as well as 3-D cultures, such as organoids.

## Microfluidic devices, including organ-on-a-chip

Microfluidic technology is a powerful tool that is well suited to establishing cultures that recapitulate different physiological compartments as well as establishing multicellular co-cultures with controlled and reproducible complexity (Osaki et al., 2018a). In its most simple form, microfluidic devices consist of two distinct compartments connected by microchannels. Basic motor circuits are generated by culturing MNs in one compartment, whose axons grow through the microchannels to innervate skeletal muscle in the other compartment after 28 days (Stoklund Dittlau et al., 2021) or 36 days (Bellmann et al., 2019). Rabies tracing, calcium imaging, and/or patch-clamp recordings are essential to establish the function of the motor circuits. Ionescu and colleagues provided procedures to measure axon growth and to reliably quantify neuromuscular junction (NMJ) activity using imaging of both muscle contractions and fast intracellular calcium changes (Ionescu et al., 2016). This platform allows precise control, monitoring, and manipulation of subcellular microenvironments. Specifically, it enables the distinction of local from retrograde signaling mechanisms and allows restricted experimental intervention in local compartments along the muscle–neuron route. In another study, Lin et al. (2020) and colleagues designed an *in vitro* NMJ induction system constructed by differentiating human iPSCs in a single culture dish. By switching the myogenic and neurogenic medium for induction, the resulting NMJ cultures contained pre- and postsynaptic components, including MNs, skeletal muscle, and Schwann cells after 1 month.

Organ-on-chip systems are more sophisticated microfluidic devices that aim to recapitulate cellular interactions, particularly among different types of cells. One particularly important structure to recapitulate is the blood-brain barrier, which has a critical function in the spinal cord and significantly impedes the delivery of many therapeutics to upper MNs. One of the advantages of microfluidic devices is the ability to control shear force and mechanical stretch as biophysical inputs (Wu et al., 2020), which could also be important contributors to MN differentiation and function. A partial 3-D blood-brain barrier model was developed with a cylindrical microchannel in collagen gel to study neuro-inflammation with a co-culture of brain endothelial cells, pericytes and astrocytes (Herland et al., 2016). Another study incorporated iPSC-derived brain-specific endothelial cells to generate a blood-brain barrier-like structure. Similarly to the blood-brain barrier *in vivo*, this *in vitro* culture reduced the therapeutic bioavailability of tested drugs (Osaki et al., 2018b).

Subsequent work successfully incorporated MNs and even generated motor units along with a model of the blood-brain barrier. Motor unit formation was successfully obtained by using microfluidic chambers where iPSC-derived MN spheroids and muscle fiber bundles were plated in different compartments of the chamber for 42 days (Osaki et al., 2018b; Osaki et al., 2018c). iPSC-derived endothelial cells mimicked the blood-brain barrier. Motor unit loss was observed using amyotrophic lateral sclerosis iPSC-derived cultures. A similar study by Sances and colleagues generated a “Spinal Cord-Chip” and demonstrated that iPSC-derived brain microvascular endothelial cells interact to promote the maturation of iPSC-derived MNs after 60 days in culture. (Sances et al., 2018).

## Organoids and assembloids

In contrast to microfluidic devices, which are synthetic devices for culturing pre-differentiated cells, iPSC-derived organoids are self-organized 3-D cultures, making them powerful tools for modeling the development of the central nervous system, including the human spinal cord. Cerebral organoids were the first human PSC-derived organoids and were generated in 2013 (Lancaster et al., 2013). They included a cerebral cortex containing progenitor population that organized and produced mature cortical neuron subtypes after 30 days in culture.

Spinal cord organoids recapitulate patterning of the developing dorsal spinal cord by SMAD inhibition followed by the addition of RA to ventralize the culture together with a SHH agonist (Lee et al., 2007; Ogura et al., 2018; Andersen et al., 2020; Valiulahi et al., 2021). Ogura and colleagues generated dorsal spinal cord-like tissues with four types of dorsal interneuron populations (Ogura et al., 2018). Increasing concentrations of a small molecule activating SHH signaling ventralized the cells, enabling the derivation of dorsal, intermediate, and ventral organoids.

In order to recapitulate a motor circuit, it is necessary to have both MNs as well as myotubes. When spinal cord organoids were co-cultured with myotubes for 35 days, NMJs formed and myotube contraction was observed, demonstrating the generation of functional motor units (Hor et al., 2018). Another study reported that iPSC-derived bipotent neuromesodermal progenitors generated trunk organoids containing spinal MNs and skeletal muscle (Faustino Martins et al., 2020). These organoids recapitulated morphogenetic movements and displayed distinct neuroectodermal and mesodermal domains. After 50 days in culture, axonal tracts innervated mature muscle fibers and formed functional NMJs.

One study generated iPSC-derived sensorimotor organoids in 105 days by producing neuromesodermal progenitors *via* FGF and WNT agonists as well as forskolin instead of using dual-SMAD inhibition (Pereira et al., 2021). iPSC-derived sensorimotor organoids cultured as free-floating sphere cultures followed by plating and growth under adherent conditions, contained sensory neurons, dorsal spinal interneurons, MNs, astrocytes, microglia, skeletal muscle, and vascular cells. Moreover, NMJs produced neuron-dependent skeletal muscle contractions, which were reduced in amyotrophic lateral sclerosis-derived organoid cultures. Furthermore, single cell sequencing revealed that spheres derived from six different isogenic iPSC lines showed minimal variation in composition.

It is particularly useful to combine different organoids into assembloids to generate more complete models of motor circuits. Upper MNs in the motor cortex innervate spinal MNs, and researchers have recapitulated this circuit by generating brain-spinal cord assembloids by co-culturing cerebral organoids and MN spheroids. Andersen and colleagues achieved one of the most complete models of MN signaling by generating a cortico-motor unit fusing cortical and spinal MNs and skeletal muscle organoids (Andersen et al., 2020). They designed a combinatorial approach (12 conditions) to test FGF-2, RA, WNT, and SHH modulators at varying concentrations following neural ectoderm specification. Following, spheroids were exposed to dual SMAD inhibition. They found that exposure to high levels of RA and low levels of FGF-2 induces an increase in rostral fates, whereas low levels of RA and high levels of FGF-2 induces an increase in caudal fates. In addition, high SAG exposure is associated with increased expression of ventral marker genes. These assembloids were maintained functionally and morphologically intact for up to 70 days *in vitro*. Using rabies tracing, calcium imaging, and patch-clamp recordings, they were shown to contain corticofugal projections and NMJs. Muscle contraction and calcium spikes in muscle fibers after optogenetic stimulation validated the functional assembly of cortico-motor units.

## Ongoing efforts to optimize organ-on-a-chip, organoids and assembloids

Compared with 2D cell culture, organoids and organ-on-a-chip have the advantages of including cell-cell and cell-extracellular matrix interaction. In the case of the microfluidic chip, there is fine control over the microenvironment. However, organ-on-a-chip are difficult to standardize and scale up, require specialized equipment (external pumps, connectors, etc.,) and are relatively expensive. Moreover, PDMS, which is commonly used to fabricate microfluidics chips can absorb molecules such as fluorescent dyes, making imaging challenging. The combination of PDMS with polytetrafluoroethylene has been proposed to reduce this problem (Yao et al., 2021). Materials such as thermoplastic polymers, elastomers and hydrogels have been suggested as alternative material for PDMS (Campbell et al., 2021).

3D cultures are heterogeneous and, unlike 2D cultures, cannot be easily integrated into existing automation systems and massively parallel screening campaigns. Compared with animal models, organoids and organ-on-a-chip could enable the study of human diseases at a lower cost with fewer ethical concerns assuming that issues of scalability and reproducibility are addressed successfully. One example are the neuromuscular organoids generated by Faustino and colleagues, which would be an ideal platform for studying NMJ diseases and identifying novel drug candidates (Faustino Martins et al., 2020).

However, there are multiple challenges when using organ-on-a-chip and organoids, including difficulties in reproducibility, resulting in considerable variability in their development, cellular composition and, in the case of organoids, organization between batches and, sometimes, organization between organoids within one experiment (Fu et al., 2021). This heterogeneity, together with the long timelines required to form and mature organoids, can make them very difficult to use and limit their application for high-throughput testing. A combination of bioengineering and biomaterial approaches can be used to minimize variability and increase reproducibility, which can be assessed using single cell RNA sequencing (Atamian et al., 2021).

Micropatterned arrays can be used as templates for generating organoids. For example, creating an artificial organizing center by embedding a mass of cells expressing a certain morphogen at one pole of the organoid (Cederquist et al., 2019) or using microfluidics (Manfrin et al., 2019; Rifes et al., 2020) or a combination of gradients in an orthogonal manner (Demers et al., 2016). Moreover, synthetic hydrogels can support organoid growth, allowing better control of biophysical and biochemical parameters (Gjorevski et al., 2016).

To reduce variability, 3D cultures need to overcome the complication of ensuring that the factors added to the culture medium are administered equally to all cells within the 3D structure. Cells within 2D cultures are widely accessible to the culture medium, enabling uniform exposure. The approach suggested by Vieira de Sá et al. (2021) and colleagues is to control the application of developmental signaling factors using microfluidic devices. iPSCs are seeded in an elongated chamber and subjected to an orthogonal gradient to generate caudal–rostral and dorsal-ventral axes for spinal cord patterning. For caudal-rostral patterning, a channel network runs on top of the main chambers connecting two inlets: the caudal inlet with FGF and an inlet with only medium creating a regulated gradient with high concentration of FGF at the caudal side and no FGF on the rostral side (Sagner & Briscoe, 2019; Rifes et al., 2020). The side chambers would deliver BMP and SHH on the top and bottom chambers for dorsoventral patterning.

Another approach is using gelatin-based 3-D bioprinted bioink scaffolds. Han et al. (2022) and colleagues bioprinted iPSC-derived MN precursors and astrocytes on different combinations of bioink that were crosslinked to scaffolds. With further optimization, this could lead to uniform spinal organoids.

Unfortunately, the lack of vascularization significantly limits the size and survival of organoids and assembloids (Lancaster et al., 2013; Grebenyuk & Ranga, 2019). As the size increases, the core becomes necrotic due to a lack of oxygen and nutrients. One strategy for addressing this problem is to fuse vascular and brain organoids to obtain vascularized brain organoids. By co-culturing primary endothelial cells, pericytes, and astrocytes, blood-brain barrier spheroids were created as an *in vitro* screening platform for brain-penetrating agents (Bergmann et al., 2018; Cho et al., 2017). Fusion organoids cultured for 40 days contained functional blood–brain barrier-like structures as well as microglial cells. Thus, in addition to potentially enabling the cultivation of larger organoids, this model also allows the modeling of interactions between neurons and non-neuronal cells, particularly the vasculature and microglia niche (Sun et al., 2022). It would be intriguing to apply this strategy to spinal cord organoids and assembloids to increase cell survival.

Another challenge is the use of brain and spinal organoids to model neurodegeneration, since organoids reach a maturational plateau that corresponds to mid-gestational stages (Camp et al., 2015; Tanaka et al., 2020). To “age” cultures, one approach uses transcription factor-mediated differentiation of a subset of PSCs into endothelial-like cells during cerebral organoid induction for 120 days, which also enhances the maturation of cells within the organoid (Cakir et al., 2019).

## Strategies to differentiate iPSCs into aged MNs

Aging is an important risk factor for neurodegenerative diseases, including MN diseases (Hou et al., 2019). Thus, for those studying neurodegeneration, the directed differentiation of iPSCs into MNs resembling those in aged spinal cord is desirable. It is important to note that aging and maturation are different in critical ways. Mature MNs manifest excitability, generate action potentials, and form functional synaptic connections as well as express synaptic markers, voltage-gated ion channels, and neurotransmitter receptors (Weick, 2016). One approach to mature iPSC-derived MNs is to transplant them into rodents to let them mature *in vivo*. After transplantation into adult mice, mES-derived MNs formed functional NMJs, a feature of mature MNs (Yohn et al., 2008). Moreover, human iPSC-derived MNs developed mature phenotypes after transplantation into mice (Corti et al., 2012; Su et al., 2013; Toma et al., 2015). Olmsted et al. (2022) and colleagues evaluated the survival and integration of transplanted iPSC-derived spinal MNs and oligodendrocyte progenitor cells in rats. Although transplanted spinal MNs expressed markers of mature neurons, aging markers were missing.

**FIGURE 3:**
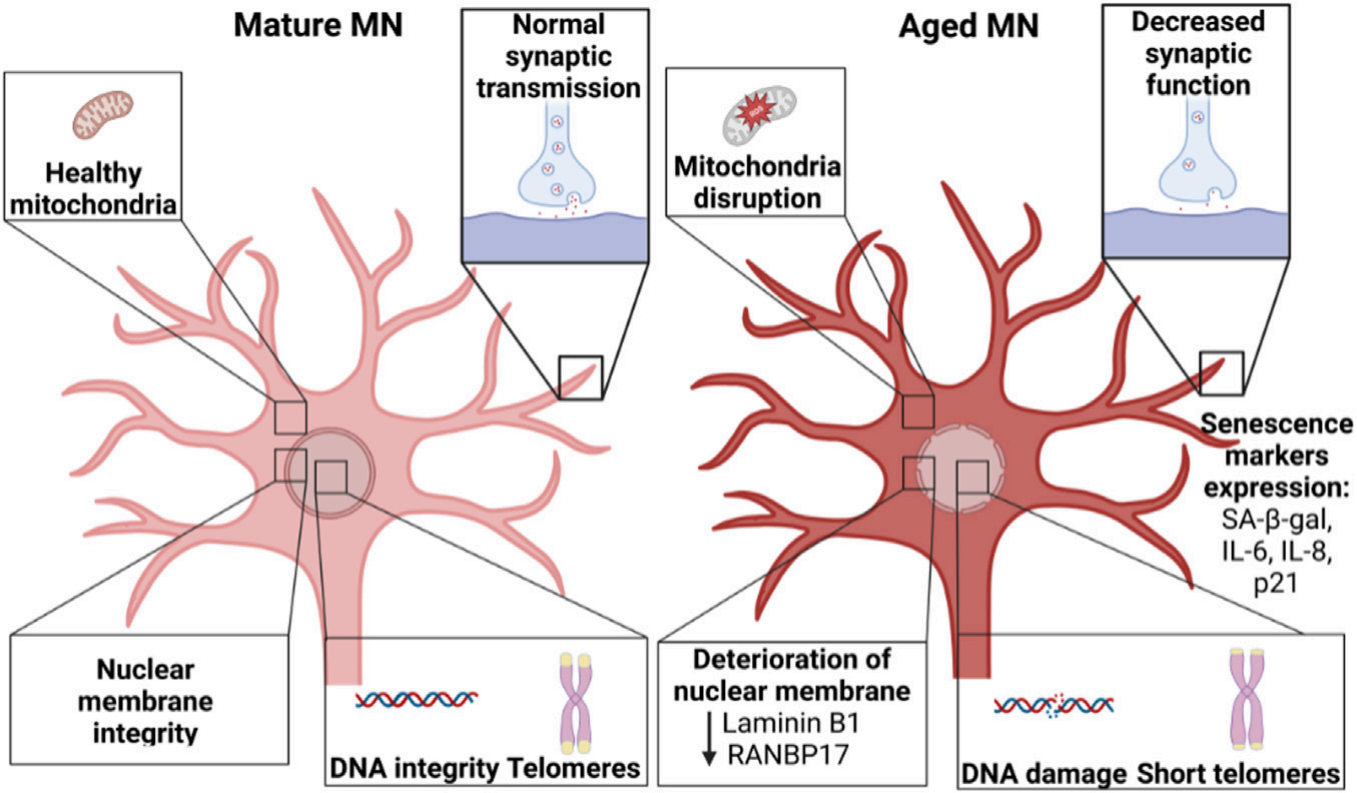
Comparison between mature MNs and aged MNs. “Aged” MNs show considerable differences compared to “young” MNs, including changes in mitochondria, synaptic function, integrity in the nuclear membrane integrity as well as increased DNA damage and reduced telomere length. Created with BioRender.com.

“Aged” neurons manifest a gradual decline in structure and function (Studer et al., 2015). In addition, hallmarks for cellular aging include shortened telomere length, reduced mitochondrial function, increased DNA damage, global reduction in heterochromatin, accumulation of damaged proteins, nuclear lamina-associated changes, and senescence (Figure 3) (Lopez-Otin et al., 2013; Miller et al., 2013). Unfortunately, reprogramming “rejuvenates” the age of somatic cells, increasing telomere length (Marion et al., 2009; Suhr et al., 2009; Agarwal et al., 2010), reducing mitochondrial disruption (Suhr et al., 2010; Prigione et al., 2011), reducing DNA damage (Miller et al., 2013), and inhibiting senescence (Lapasset et al., 2011). As a result, iPSC-derived neurons resemble fetal neurons rather than those of the aged neurons of the donating patient (Mariani et al., 2012; Nicholas et al., 2013). Thus, it is important to assess regularly iPSC-derived MNs for markers of maturation and function as well as “aging”. Possible assays for testing iPSC-derived MNs include: senescence markers such as p21 and beta-galactosidase, telomere length measurement, nuclear lamina changes, and mitochondrial function in addition to functional assays such as electrophysiology and synaptogenesis. Comparing the transcriptome of iPSC-derived MNs against *in vivo* MNs could also be very valuable.

Premature aging diseases such as Hutchinson–Gilford progeria syndrome suggest strategies to artificially “age” iPSC-derived neurons. Progeria caused by Progerin is a truncated version of the lamin A protein (Scaffidi & Misteli, 2005). Progerin induces structural and functional changes in the nuclear membrane, leading to damage, telomere shortening, p53-dependent changes in gene expression regulation, and induction of cellular senescence and cell death (Dechat et al., 2008). Expression of progerin in iPSC-derived midbrain dopaminergic neurons resulted in aged-specific characteristics, including increased DNA damage and mitochondrial dysfunction, reduced dendrite length, increased neuromelanin production, apoptosis, mitochondrial defects, and neuronal inclusions indicative of decreased ubiquitin-proteasome function (Miller et al., 2013). It might prove interesting to use a similar strategy to artificially “age” iPSC-derived MNs. Nevertheless, it is important to note that progerin-induced changes occur *via* a different molecular mechanism in comparison to normal aging (Brennand, 2013).

Another potential approach to induce “aging” is inhibition of telomerase, an enzyme that causes telomere elongation. Telomeres are special nucleoprotein structures at the ends of eukaryotic chromosomes that protect them from degradation and DNA damage, and telomeres shorten with aging (Aubert & Lansdorp, 2008). It has been shown that inhibiting telomerase in iPSCs using BIBR1532 followed by differentiation into midbrain dopaminergic neurons resulted in a manifestation of age-related phenotypes such as DNA damage, dendritic atrophy and mitochondrial perturbation (Vera et al., 2016). Unfortunately, iPSCs and iPSC-derived MNs treated with BIBR1532 either did not survive, or, at low and well-tolerated concentrations, did not manifest age-related phenotypes (Pandya et al., 2021). Thus, this approach might require further optimization, such as testing increasing treatment durations. Alternatively, it is possible that a different telomerase inhibitor, such as U-73122, purpuromycin, beta-rubromycin, and epigallocatechin gallate (Shalaby et al., 2010), might be more successful than BIBR1532.

Finally, aging is associated with specific changes in gene expression, including for the genes encoding RANBP17 and NRSF/REST. Thus, it might be possible to artificially “age” iPSC-derived MNs by manipulating the expression of age-associated proteins such as RANBP17 and NRSF/REST. For example, Mertens and colleagues showed that iPSCs are “rejuvenated” during reprogramming. They compared neurons from human donors across a broad range of ages, either by differentiation from iPSCs or by direct conversion into induced neurons from fibroblasts (iNs). iPSC-derived neurons did not retain aging-associated gene signatures. In contrast, iNs displayed age-specific transcriptional profiles and revealed age-associated decreases in the nuclear transport receptor RANBP17. Using RNA sequencing, they showed that RANBP17 levels decreased with age (Mertens et al., 2021). This could suggest that reducing the expression of RANBP17, for example *via* knock-down, could artificially “age” iPSC-derived MNs.

Interestingly, it is possible that disease might be associated with aberrant expression of proteins required for healthy aging. For example, NRSF/REST is a feature of normal aging in human cortical and hippocampal neurons; its expression is low in “young” neurons and high in “aged” neurons (Lu et al., 2014). However, iPSC-derived neurons from sporadic Alzheimer’s disease patients show reduced NRSF/REST and was associated with disruption of the nuclear lamina (Meyer et al., 2019). This suggests that “healthy” aging mechanisms are dysregulated in neurodegenerative diseases, such as Alzheimer’s disease. To our knowledge, there is no study measuring NRSF/REST levels in MNs in young vs. aged spinal cord or in iPSC-derived MNs.

## Concluding remarks

iPSCs are powerful tools for modeling MN diseases, but doing so requires large quantities of functional MNs having the same characteristics, age, and functional connections as those found in patients. There has been considerable success in developing protocols to direct the differentiation of iPSCs into MNs, which can now be obtained near homogeneity within 1–2 weeks. Recent years, have seen a considerable increase in the diversity and sophistication of MN cultures, each marking an improvement in replicating human MNs and physiological function *in vivo*. As summarized in Table 3, all of the approaches have advantages and disadvantages. Thus, the selected model system must fit the research question under investigation. For example, pure and specific subtypes of MN cultures, using either small molecules or transcription factor expression, enable the study of developing MNs, cell-autonomous disease mechanisms and are easily scaled up for high-throughput screening or transplantation. Meanwhile, microfluidics and organoids are well suited to studying non-cell autonomous pathogenesis and assessing MN function, for example, using myotube contraction. Considerable effort is being made to reduce variability in order to increase the number and types of experiments in which iPSC-derived MNs can be used. Finally, it remains challenging to obtain iPSC-derived MNs showing age-associated pathogenesis, but strategies are being developed that could facilitate the artificial “aging” of iPSC-derived MNs *in vitro*.

**TABLE 3:** Advantages and disadvantages of different approaches to obtain MNs in culture.

	Small molecules	Transcription factors	Organ-on-chip	Organoids
Duration	Long term	Short term	Intermediate term	Long term
MN purity	Variable	High	Variable	Variable
Cost	Relative cheap	Relative cheap	Expensive	Expensive
Reproducibility	High variability	Low variability	Low variability	High variability
Maturation features	Absence of mature feature as single culture	Absence of mature feature as single culture	Improve of MN maturation in co-culture	Improve of MN maturation
Applicability	Drug screening	Drug screening	Drug screening including toxicity	Mechanism of cell-cell interaction
	Mechanism of a single cell	Mechanism of a single cell	Mechanism of cell-cell interaction	
Scalability	Easy to scale up	Easy to scale up	Difficult to scale up	Difficult to scale up
Manipulation	Easy to handle	Easy to handle	Proper training needed	Proper training needed
